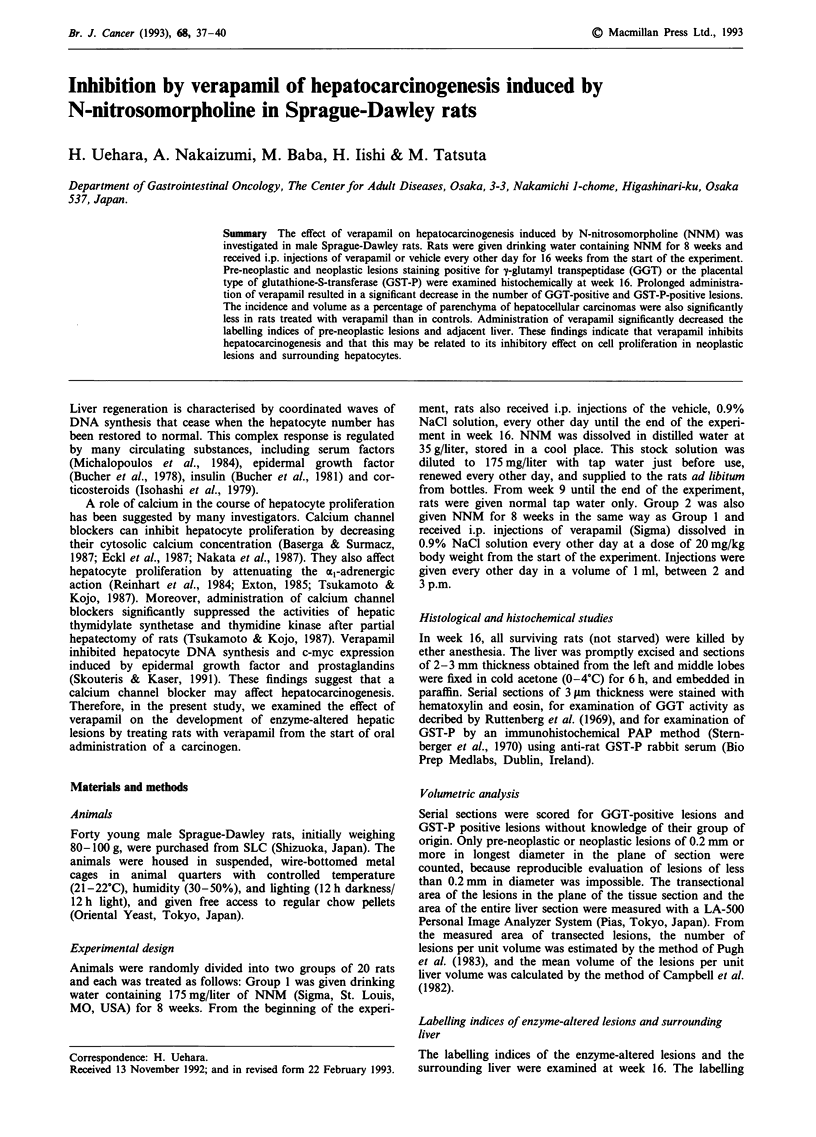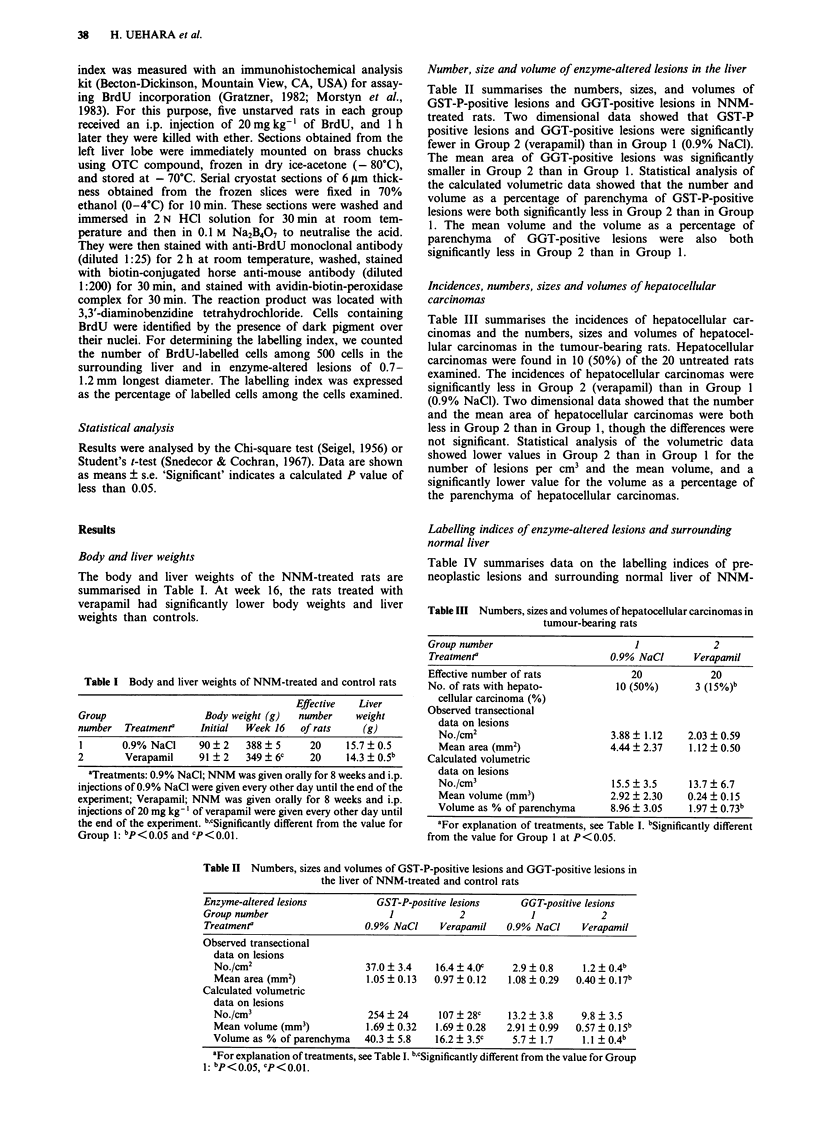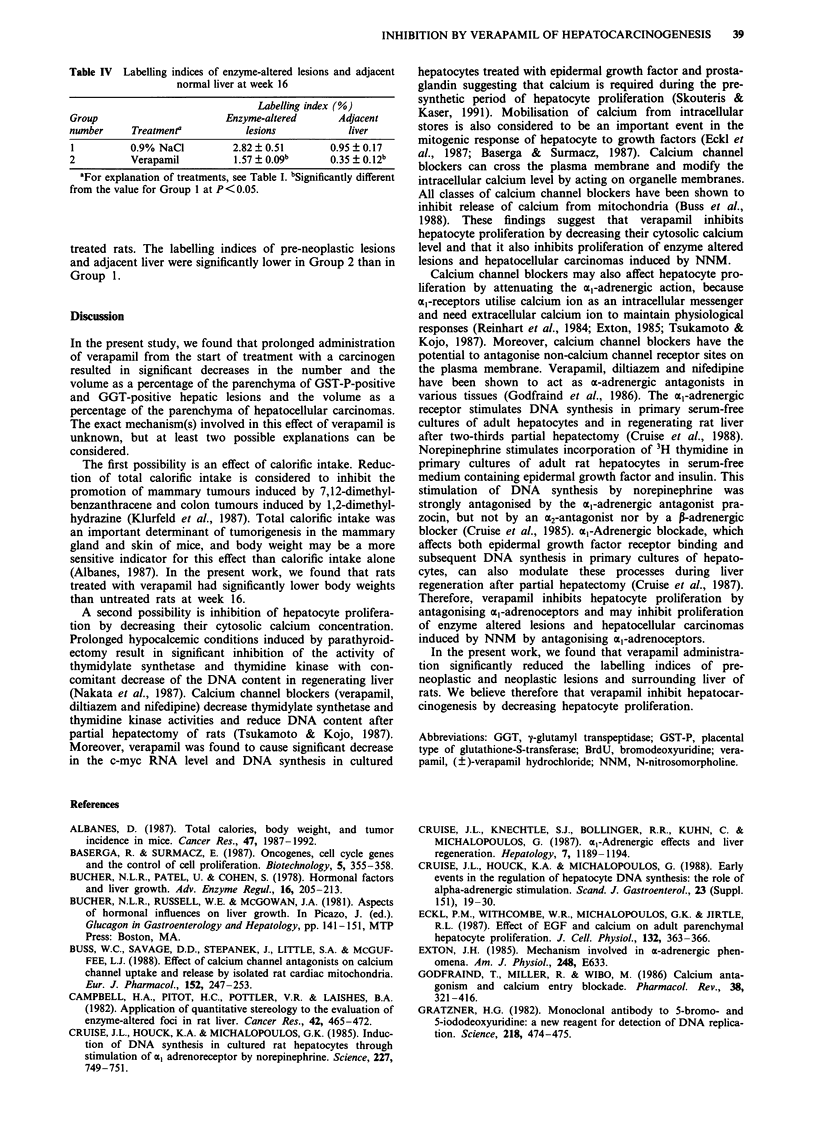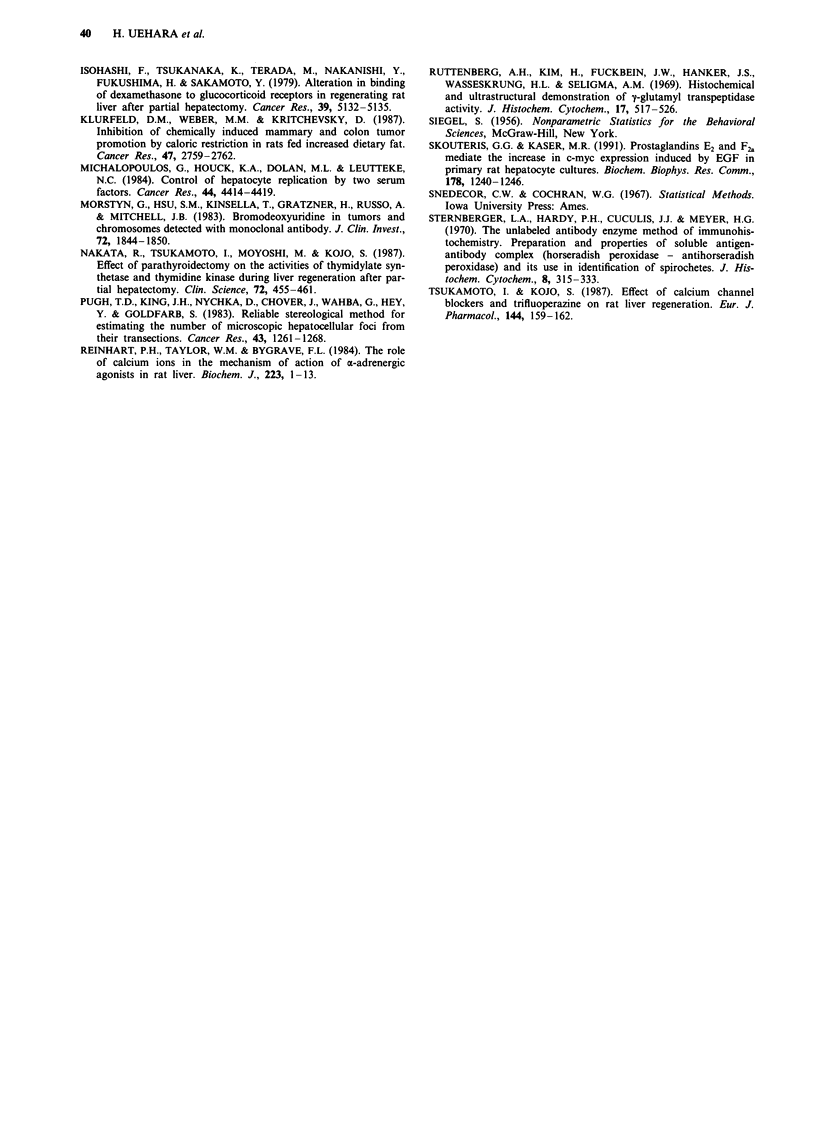# Inhibition by verapamil of hepatocarcinogenesis induced by N-nitrosomorpholine in Sprague-Dawley rats.

**DOI:** 10.1038/bjc.1993.283

**Published:** 1993-07

**Authors:** H. Uehara, A. Nakaizumi, M. Baba, H. Iishi, M. Tatsuta

**Affiliations:** Department of Gastrointestinal Oncology, Center for Adult Diseases, Osaka, Japan.

## Abstract

The effect of verapamil on hepatocarcinogenesis induced by N-nitrosomorpholine (NNM) was investigated in male Sprague-Dawley rats. Rats were given drinking water containing NNM for 8 weeks and received i.p. injections of verapamil or vehicle every other day for 16 weeks from the start of the experiment. Pre-neoplastic and neoplastic lesions staining positive for gamma-glutamyl transpeptidase (GGT) or the placental type of glutathione-S-transferase (GST-P) were examined histochemically at week 16. Prolonged administration of verapamil resulted in a significant decrease in the number of GGT-positive and GST-P-positive lesions. The incidence and volume as a percentage of parenchyma of hepatocellular carcinomas were also significantly less in rats treated with verapamil than in controls. Administration of verapamil significantly decreased the labelling indices of pre-neoplastic lesions and adjacent liver. These findings indicate that verapamil inhibits hepatocarcinogenesis and that this may be related to its inhibitory effect on cell proliferation in neoplastic lesions and surrounding hepatocytes.


*Br. J. Cancer* (1993), **68**, 37–40 © Macmillan Press Ltd., 1993

### Inhibition by verapamil of hepatocarcinogenesis induced by N-nitrosomorpholine in Sprague-Dawley rats

H. Uehara, A. Nakaizumi, M. Baba, H. Iishi & M. Tatsuta

*Department of Gastrointestinal Oncology, The Center for Adult Diseases, Osaka, 3-3, Nakamichi 1-chome, Higashinari-ku, Osaka 537, Japan.*

**Summary** The effect of verapamil on hepatocarcinogenesis induced by N-nitrosomorpholine (NNM) was investigated in male Sprague-Dawley rats. Rats were given drinking water containing NNM for 8 weeks and received i.p. injections of verapamil or vehicle every other day for 16 weeks from the start of the experiment. Pre-neoplastic and neoplastic lesions staining positive for γ-glutamyl transpeptidase (GGT) or the placental type of glutathione-S-transferase (GST-P) were examined histochemically at week 16. Prolonged administration of verapamil resulted in a significant decrease in the number of GGT-positive and GST-P-positive lesions. The incidence and volume as a percentage of parenchyma of hepatocellular carcinomas were also significantly less in rats treated with verapamil than in controls. Administration of verapamil significantly decreased the labelling indices of pre-neoplastic lesions and adjacent liver. These findings indicate that verapamil inhibits hepatocarcinogenesis and that this may be related to its inhibitory effect on cell proliferation in neoplastic lesions and surrounding hepatocytes.

Liver regeneration is characterised by coordinated waves of DNA synthesis that cease when the hepatocyte number has been restored to normal. This complex response is regulated by many circulating substances, including serum factors (Michalopoulos *et al.*, 1984), epidermal growth factor (Bucher *et al.*, 1978), insulin (Bucher *et al.*, 1981) and corticosteroids (Isohashi *et al.*, 1979).

A role of calcium in the course of hepatocyte proliferation has been suggested by many investigators. Calcium channel blockers can inhibit hepatocyte proliferation by decreasing their cytosolic calcium concentration (Baserga & Surmacz, 1987; Eckl *et al.*, 1987; Nakata *et al.*, 1987). They also affect hepatocyte proliferation by attenuating the $\alpha_1$-adrenergic action (Reinhart *et al.*, 1984; Exton, 1985; Tsukamoto & Kojo, 1987). Moreover, administration of calcium channel blockers significantly suppressed the activities of hepatic thymidylate synthetase and thymidine kinase after partial hepatectomy of rats (Tsukamoto & Kojo, 1987). Verapamil inhibited hepatocyte DNA synthesis and c-myc expression induced by epidermal growth factor and prostaglandins (Skouteris & Kaser, 1991). These findings suggest that a calcium channel blocker may affect hepatocarcinogenesis. Therefore, in the present study, we examined the effect of verapamil on the development of enzyme-altered hepatic lesions by treating rats with verapamil from the start of oral administration of a carcinogen.

### Materials and methods

### *Animals*

Forty young male Sprague-Dawley rats, initially weighing 80–100 g, were purchased from SLC (Shizuoka, Japan). The animals were housed in suspended, wire-bottomed metal cages in animal quarters with controlled temperature (21–22°C), humidity (30–50%), and lighting (12 h darkness/12 h light), and given free access to regular chow pellets (Oriental Yeast, Tokyo, Japan).

### *Experimental design*

Animals were randomly divided into two groups of 20 rats and each was treated as follows: Group 1 was given drinking water containing 175 mg/liter of NNM (Sigma, St. Louis, MO, USA) for 8 weeks. From the beginning of the experiment, rats also received i.p. injections of the vehicle, 0.9% NaCl solution, every other day until the end of the experiment in week 16. NNM was dissolved in distilled water at 35 g/liter, stored in a cool place. This stock solution was diluted to 175 mg/liter with tap water just before use, renewed every other day, and supplied to the rats *ad libitum* from bottles. From week 9 until the end of the experiment, rats were given normal tap water only. Group 2 was also given NNM for 8 weeks in the same way as Group 1 and received i.p. injections of verapamil (Sigma) dissolved in 0.9% NaCl solution every other day at a dose of 20 mg/kg body weight from the start of the experiment. Injections were given every other day in a volume of 1 ml, between 2 and 3 p.m.

### *Histological and histochemical studies*

In week 16, all surviving rats (not starved) were killed by ether anesthesia. The liver was promptly excised and sections of 2–3 mm thickness obtained from the left and middle lobes were fixed in cold acetone (0–4°C) for 6 h, and embedded in paraffin. Serial sections of 3 μm thickness were stained with hematoxylin and eosin, for examination of GGT activity as decribed by Ruttenberg *et al.* (1969), and for examination of GST-P by an immunohistochemical PAP method (Sternberger *et al.*, 1970) using anti-rat GST-P rabbit serum (Bio Prep Medlabs, Dublin, Ireland).

### *Volumetric analysis*

Serial sections were scored for GGT-positive lesions and GST-P positive lesions without knowledge of their group of origin. Only pre-neoplastic or neoplastic lesions of 0.2 mm or more in longest diameter in the plane of section were counted, because reproducible evaluation of lesions of less than 0.2 mm in diameter was impossible. The transectional area of the lesions in the plane of the tissue section and the area of the entire liver section were measured with a LA-500 Personal Image Analyzer System (Pias, Tokyo, Japan). From the measured area of transected lesions, the number of lesions per unit volume was estimated by the method of Pugh *et al.* (1983), and the mean volume of the lesions per unit liver volume was calculated by the method of Campbell *et al.* (1982).

### *Labelling indices of enzyme-altered lesions and surrounding liver*

The labelling indices of the enzyme-altered lesions and the surrounding liver were examined at week 16. The labelling

Correspondence: H. Uehara.
Received 13 November 1992; and in revised form 22 February 1993.

index was measured with an immunohistochemical analysis kit (Becton-Dickinson, Mountain View, CA, USA) for assaying BrdU incorporation (Gratzner, 1982; Morstyn et al., 1983). For this purpose, five unstarved rats in each group received an i.p. injection of 20 mg $kg^{-1}$ of BrdU, and 1 h later they were killed with ether. Sections obtained from the left liver lobe were immediately mounted on brass chucks using OTC compound, frozen in dry ice-acetone (– 80°C), and stored at – 70°C. Serial cryostat sections of 6 µm thickness obtained from the frozen slices were fixed in 70% ethanol (0–4°C) for 10 min. These sections were washed and immersed in 2 N HCl solution for 30 min at room temperature and then in 0.1 M $Na_2B_4O_7$ to neutralise the acid. They were then stained with anti-BrdU monoclonal antibody (diluted 1:25) for 2 h at room temperature, washed, stained with biotin-conjugated horse anti-mouse antibody (diluted 1:200) for 30 min, and stained with avidin-biotin-peroxidase complex for 30 min. The reaction product was located with 3,3′-diaminobenzidine tetrahydrochloride. Cells containing BrdU were identified by the presence of dark pigment over their nuclei. For determining the labelling index, we counted the number of BrdU-labelled cells among 500 cells in the surrounding liver and in enzyme-altered lesions of 0.7–1.2 mm longest diameter. The labelling index was expressed as the percentage of labelled cells among the cells examined.

*Statistical analysis*

Results were analysed by the Chi-square test (Seigel, 1956) or Student's *t*-test (Snedecor & Cochran, 1967). Data are shown as means ± s.e. 'Significant' indicates a calculated *P* value of less than 0.05.

### Results

*Body and liver weights*

The body and liver weights of the NNM-treated rats are summarised in Table I. At week 16, the rats treated with verapamil had significantly lower body weights and liver weights than controls.

**Table I** Body and liver weights of NNM-treated and control rats

| Group number | Treatment[a] | Body weight (g) Initial | Body weight (g) Week 16 | Effective number of rats | Liver weight (g) |
|---|---|---|---|---|---|
| 1 | 0.9% NaCl | 90 ± 2 | 388 ± 5 | 20 | 15.7 ± 0.5 |
| 2 | Verapamil | 91 ± 2 | 349 ± 6[c] | 20 | 14.3 ± 0.5[b] |

[a]Treatments: 0.9% NaCl; NNM was given orally for 8 weeks and i.p. injections of 0.9% NaCl were given every other day until the end of the experiment; Verapamil; NNM was given orally for 8 weeks and i.p. injections of 20 mg $kg^{-1}$ of verapamil were given every other day until the end of the experiment. [b,c]Significantly different from the value for Group 1: [b]$P<0.05$ and [c]$P<0.01$.

*Number, size and volume of enzyme-altered lesions in the liver*

Table II summarises the numbers, sizes, and volumes of GST-P-positive lesions and GGT-positive lesions in NNM-treated rats. Two dimensional data showed that GST-P positive lesions and GGT-positive lesions were significantly fewer in Group 2 (verapamil) than in Group 1 (0.9% NaCl). The mean area of GGT-positive lesions was significantly smaller in Group 2 than in Group 1. Statistical analysis of the calculated volumetric data showed that the number and volume as a percentage of parenchyma of GST-P-positive lesions were both significantly less in Group 2 than in Group 1. The mean volume and the volume as a percentage of parenchyma of GGT-positive lesions were also both significantly less in Group 2 than in Group 1.

*Incidences, numbers, sizes and volumes of hepatocellular carcinomas*

Table III summarises the incidences of hepatocellular carcinomas and the numbers, sizes and volumes of hepatocellular carcinomas in the tumour-bearing rats. Hepatocellular carcinomas were found in 10 (50%) of the 20 untreated rats examined. The incidences of hepatocellular carcinomas were significantly less in Group 2 (verapamil) than in Group 1 (0.9% NaCl). Two dimensional data showed that the number and the mean area of hepatocellular carcinomas were both less in Group 2 than in Group 1, though the differences were not significant. Statistical analysis of the volumetric data showed lower values in Group 2 than in Group 1 for the number of lesions per $cm^3$ and the mean volume, and a significantly lower value for the volume as a percentage of the parenchyma of hepatocellular carcinomas.

*Labelling indices of enzyme-altered lesions and surrounding normal liver*

Table IV summarises data on the labelling indices of preneoplastic lesions and surrounding normal liver of NNM-

**Table III** Numbers, sizes and volumes of hepatocellular carcinomas in tumour-bearing rats

| Group number<br>Treatment[a] | 1<br>0.9% NaCl | 2<br>Verapamil |
|---|---|---|
| Effective number of rats | 20 | 20 |
| No. of rats with hepatocellular carcinoma (%) | 10 (50%) | 3 (15%)[b] |
| Observed transectional data on lesions | | |
| No./$cm^2$ | 3.88 ± 1.12 | 2.03 ± 0.59 |
| Mean area ($mm^2$) | 4.44 ± 2.37 | 1.12 ± 0.50 |
| Calculated volumetric data on lesions | | |
| No./$cm^3$ | 15.5 ± 3.5 | 13.7 ± 6.7 |
| Mean volume ($mm^3$) | 2.92 ± 2.30 | 0.24 ± 0.15 |
| Volume as % of parenchyma | 8.96 ± 3.05 | 1.97 ± 0.73[b] |

[a]For explanation of treatments, see Table I. [b]Significantly different from the value for Group 1 at $P<0.05$.

**Table II** Numbers, sizes and volumes of GST-P-positive lesions and GGT-positive lesions in the liver of NNM-treated and control rats

| Enzyme-altered lesions | GST-P-positive lesions | | GGT-positive lesions | |
|---|---|---|---|---|
| Group number | 1 | 2 | 1 | 2 |
| Treatment[a] | 0.9% NaCl | Verapamil | 0.9% NaCl | Verapamil |
| Observed transectional data on lesions | | | | |
| No./$cm^2$ | 37.0 ± 3.4 | 16.4 ± 4.0[c] | 2.9 ± 0.8 | 1.2 ± 0.4[b] |
| Mean area ($mm^2$) | 1.05 ± 0.13 | 0.97 ± 0.12 | 1.08 ± 0.29 | 0.40 ± 0.17[b] |
| Calculated volumetric data on lesions | | | | |
| No./$cm^3$ | 254 ± 24 | 107 ± 28[c] | 13.2 ± 3.8 | 9.8 ± 3.5 |
| Mean volume ($mm^3$) | 1.69 ± 0.32 | 1.69 ± 0.28 | 2.91 ± 0.99 | 0.57 ± 0.15[b] |
| Volume as % of parenchyma | 40.3 ± 5.8 | 16.2 ± 3.5[c] | 5.7 ± 1.7 | 1.1 ± 0.4[b] |

[a]For explanation of treatments, see Table I. [b,c]Significantly different from the value for Group 1: [b]$P<0.05$, [c]$P<0.01$.

**Table IV** Labelling indices of enzyme-altered lesions and adjacent normal liver at week 16

| Group number | Treatment[a] | Labelling index (%) Enzyme-altered lesions | Adjacent liver |
|---|---|---|---|
| 1 | 0.9% NaCl | 2.82 ± 0.51 | 0.95 ± 0.17 |
| 2 | Verapamil | 1.57 ± 0.09[b] | 0.35 ± 0.12[b] |

[a]For explanation of treatments, see Table I. [b]Significantly different from the value for Group 1 at $P<0.05$.

treated rats. The labelling indices of pre-neoplastic lesions and adjacent liver were significantly lower in Group 2 than in Group 1.

### Discussion

In the present study, we found that prolonged administration of verapamil from the start of treatment with a carcinogen resulted in significant decreases in the number and the volume as a percentage of the parenchyma of GST-P-positive and GGT-positive hepatic lesions and the volume as a percentage of the parenchyma of hepatocellular carcinomas. The exact mechanism(s) involved in this effect of verapamil is unknown, but at least two possible explanations can be considered.

The first possibility is an effect of calorific intake. Reduction of total calorific intake is considered to inhibit the promotion of mammary tumours induced by 7,12-dimethylbenzanthracene and colon tumours induced by 1,2-dimethylhydrazine (Klurfeld *et al.*, 1987). Total calorific intake was an important determinant of tumorigenesis in the mammary gland and skin of mice, and body weight may be a more sensitive indicator for this effect than calorific intake alone (Albanes, 1987). In the present work, we found that rats treated with verapamil had significantly lower body weights than untreated rats at week 16.

A second possibility is inhibition of hepatocyte proliferation by decreasing their cytosolic calcium concentration. Prolonged hypocalcemic conditions induced by parathyroidectomy result in significant inhibition of the activity of thymidylate synthetase and thymidine kinase with concomitant decrease of the DNA content in regenerating liver (Nakata *et al.*, 1987). Calcium channel blockers (verapamil, diltiazem and nifedipine) decrease thymidylate synthetase and thymidine kinase activities and reduce DNA content after partial hepatectomy of rats (Tsukamoto & Kojo, 1987). Moreover, verapamil was found to cause significant decrease in the c-myc RNA level and DNA synthesis in cultured hepatocytes treated with epidermal growth factor and prostaglandin suggesting that calcium is required during the presynthetic period of hepatocyte proliferation (Skouteris & Kaser, 1991). Mobilisation of calcium from intracellular stores is also considered to be an important event in the mitogenic response of hepatocyte to growth factors (Eckl *et al.*, 1987; Baserga & Surmacz, 1987). Calcium channel blockers can cross the plasma membrane and modify the intracellular calcium level by acting on organelle membranes. All classes of calcium channel blockers have been shown to inhibit release of calcium from mitochondria (Buss *et al.*, 1988). These findings suggest that verapamil inhibits hepatocyte proliferation by decreasing their cytosolic calcium level and that it also inhibits proliferation of enzyme altered lesions and hepatocellular carcinomas induced by NNM.

Calcium channel blockers may also affect hepatocyte proliferation by attenuating the $\alpha_1$-adrenergic action, because $\alpha_1$-receptors utilise calcium ion as an intracellular messenger and need extracellular calcium ion to maintain physiological responses (Reinhart *et al.*, 1984; Exton, 1985; Tsukamoto & Kojo, 1987). Moreover, calcium channel blockers have the potential to antagonise non-calcium channel receptor sites on the plasma membrane. Verapamil, diltiazem and nifedipine have been shown to act as $\alpha$-adrenergic antagonists in various tissues (Godfraind *et al.*, 1986). The $\alpha_1$-adrenergic receptor stimulates DNA synthesis in primary serum-free cultures of adult hepatocytes and in regenerating rat liver after two-thirds partial hepatectomy (Cruise *et al.*, 1988). Norepinephrine stimulates incorporation of $^3$H thymidine in primary cultures of adult rat hepatocytes in serum-free medium containing epidermal growth factor and insulin. This stimulation of DNA synthesis by norepinephrine was strongly antagonised by the $\alpha_1$-adrenergic antagonist prazocin, but not by an $\alpha_2$-antagonist nor by a $\beta$-adrenergic blocker (Cruise *et al.*, 1985). $\alpha_1$-Adrenergic blockade, which affects both epidermal growth factor receptor binding and subsequent DNA synthesis in primary cultures of hepatocytes, can also modulate these processes during liver regeneration after partial hepatectomy (Cruise *et al.*, 1987). Therefore, verapamil inhibits hepatocyte proliferation by antagonising $\alpha_1$-adrenoceptors and may inhibit proliferation of enzyme altered lesions and hepatocellular carcinomas induced by NNM by antagonising $\alpha_1$-adrenoceptors.

In the present work, we found that verapamil administration significantly reduced the labelling indices of preneoplastic and neoplastic lesions and surrounding liver of rats. We believe therefore that verapamil inhibit hepatocarcinogenesis by decreasing hepatocyte proliferation.

Abbreviations: GGT, $\gamma$-glutamyl transpeptidase; GST-P, placental type of glutathione-S-transferase; BrdU, bromodeoxyuridine; verapamil, (±)-verapamil hydrochloride; NNM, N-nitrosomorpholine.

### References

ALBANES, D. (1987). Total calories, body weight, and tumor incidence in mice. *Cancer Res.*, **47**, 1987-1992.

BASERGA, R. & SURMACZ, E. (1987). Oncogenes, cell cycle genes and the control of cell proliferation. *Biotechnology*, **5**, 355-358.

BUCHER, N.L.R., PATEL, U. & COHEN, S. (1978). Hormonal factors and liver growth. *Adv. Enzyme Regul.*, **16**, 205-213.

BUCHER, N.L.R., RUSSELL, W.E. & MCGOWAN, J.A. (1981). Aspects of hormonal influences on liver growth. In Picazo, J. (ed.). *Glucagon in Gastroenterology and Hepatology*, pp. 141-151, MTP Press: Boston, MA.

BUSS, W.C., SAVAGE, D.D., STEPANEK, J., LITTLE, S.A. & MCGUFFEE, L.J. (1988). Effect of calcium channel antagonists on calcium channel uptake and release by isolated rat cardiac mitochondria. *Eur. J. Pharmacol.*, **152**, 247-253.

CAMPBELL, H.A., PITOT, H.C., POTTLER, V.R. & LAISHES, B.A. (1982). Application of quantitative stereology to the evaluation of enzyme-altered foci in rat liver. *Cancer Res.*, **42**, 465-472.

CRUISE, J.L., HOUCK, K.A. & MICHALOPOULOS, G.K. (1985). Induction of DNA synthesis in cultured rat hepatocytes through stimulation of $\alpha_1$ adrenoreceptor by norepinephrine. *Science*, **227**, 749-751.

CRUISE, J.L., KNECHTLE, S.J., BOLLINGER, R.R., KUHN, C. & MICHALOPOULOS, G. (1987). $\alpha_1$-Adrenergic effects and liver regeneration. *Hepatology*, **7**, 1189-1194.

CRUISE, J.L., HOUCK, K.A. & MICHALOPOULOS, G. (1988). Early events in the regulation of hepatocyte DNA synthesis: the role of alpha-adrenergic stimulation. *Scand. J. Gastroenterol.*, **23** (Suppl. 151), 19-30.

ECKL, P.M., WITHCOMBE, W.R., MICHALOPOULOS, G.K. & JIRTLE, R.L. (1987). Effect of EGF and calcium on adult parenchymal hepatocyte proliferation. *J. Cell. Physiol.*, **132**, 363-366.

EXTON, J.H. (1985). Mechanism involved in $\alpha$-adrenergic phenomena. *Am. J. Physiol.*, **248**, E633.

GODFRAIND, T., MILLER, R. & WIBO, M. (1986) Calcium antagonism and calcium entry blockade. *Pharmacol. Rev.*, **38**, 321-416.

GRATZNER, H.G. (1982). Monoclonal antibody to 5-bromo- and 5-iododeoxyuridine: a new reagent for detection of DNA replication. *Science*, **218**, 474-475.

ISOHASHI, F., TSUKANAKA, K., TERADA, M., NAKANISHI, Y., FUKUSHIMA, H. & SAKAMOTO, Y. (1979). Alteration in binding of dexamethasone to glucocorticoid receptors in regenerating rat liver after partial hepatectomy. *Cancer Res.*, **39**, 5132–5135.

KLURFELD, D.M., WEBER, M.M. & KRITCHEVSKY, D. (1987). Inhibition of chemically induced mammary and colon tumor promotion by caloric restriction in rats fed increased dietary fat. *Cancer Res.*, **47**, 2759–2762.

MICHALOPOULOS, G., HOUCK, K.A., DOLAN, M.L. & LEUTTEKE, N.C. (1984). Control of hepatocyte replication by two serum factors. *Cancer Res.*, **44**, 4414–4419.

MORSTYN, G., HSU, S.M., KINSELLA, T., GRATZNER, H., RUSSO, A. & MITCHELL, J.B. (1983). Bromodeoxyuridine in tumors and chromosomes detected with monoclonal antibody. *J. Clin. Invest.*, **72**, 1844–1850.

NAKATA, R., TSUKAMOTO, I., MOYOSHI, M. & KOJO, S. (1987). Effect of parathyroidectomy on the activities of thymidylate synthetase and thymidine kinase during liver regeneration after partial hepatectomy. *Clin. Science*, **72**, 455–461.

PUGH, T.D., KING, J.H., NYCHKA, D., CHOVER, J., WAHBA, G., HEY, Y. & GOLDFARB, S. (1983). Reliable stereological method for estimating the number of microscopic hepatocellular foci from their transections. *Cancer Res.*, **43**, 1261–1268.

REINHART, P.H., TAYLOR, W.M. & BYGRAVE, F.L. (1984). The role of calcium ions in the mechanism of action of α-adrenergic agonists in rat liver. *Biochem. J.*, **223**, 1–13.

RUTTENBERG, A.H., KIM, H., FUCKBEIN, J.W., HANKER, J.S., WASSESKRUNG, H.L. & SELIGMA, A.M. (1969). Histochemical and ultrastructural demonstration of γ-glutamyl transpeptidase activity. *J. Histochem. Cytochem.*, **17**, 517–526.

SIEGEL, S. (1956). *Nonparametric Statistics for the Behavioral Sciences*, McGraw-Hill, New York.

SKOUTERIS, G.G. & KASER, M.R. (1991). Prostaglandins $E_2$ and $F_{2a}$ mediate the increase in c-myc expression induced by EGF in primary rat hepatocyte cultures. *Biochem. Biophys. Res. Comm.*, **178**, 1240–1246.

SNEDECOR, C.W. & COCHRAN, W.G. (1967). *Statistical Methods.* Iowa University Press: Ames.

STERNBERGER, L.A., HARDY, P.H., CUCULIS, J.J. & MEYER, H.G. (1970). The unlabeled antibody enzyme method of immunohistochemistry. Preparation and properties of soluble antigen-antibody complex (horseradish peroxidase – antihorseradish peroxidase) and its use in identification of spirochetes. *J. Histochem. Cytochem.*, **8**, 315–333.

TSUKAMOTO, I. & KOJO, S. (1987). Effect of calcium channel blockers and trifluoperazine on rat liver regeneration. *Eur. J. Pharmacol.*, **144**, 159–162.